# Pneumonia and Its Treatment

**Published:** 1904-05

**Authors:** Henry R. Slack

**Affiliations:** President of the Georgia Pasteur Institute, Atlanta, and Superintendent of the LaGrange Sanatorium, LaGrange, Ga.


### PNEUMONIA AND ITS TREATMENT.

BY HENRY R. SLACK, PH.M., M.D.,

President of the Georgia Pasteur Institute, Atlanta, and Superintendent of the LaGrange Sanatorium, LaGrange, Ga.

Consumption was the undisputed "Captain of the Host of Death" until 1890. The brilliant discovery of Koch in 1882 has improved our methods of battling with him, weakened his hold and lessened his sway to such an extent he may lose his command.

### MORTALITY INCREASING.

Pneumonia, his active first-lieutenant, is ambitious, and according to statistics is supplanting him in leading the host of Death in the cities. It is not pleasant to admit retrogression, but statistics and observation in the cities and hospitals force me to acknowledge that pneumonia is not as successfully treated now as it was twenty or thirty years ago. The death rate from consumption in 1900 as compared with 1880 has decreased 20.7 per cent. in proportion to popoulation, and 15.2 per cent. in proportion to total mortality. The general total mortality has in the meantime decreased 6.3 per cent. Pneumonia, on the contrary, has increased in mortality 11 per cent. in proportion to population from 1890 to 1900, and in proportion to mortality 12.21 per cent. The deaths in 10,000 of population since 1860 show for pneumonia an increase of over 300 per cent.; while for consumption a decrease of 39.5 per cent. Since 1900 pneumonia has caused one-third more deaths in Chicago than consumption, and the reports from other cities are almost as bad.

Why this increase in mortality from pneumonia, when there has been a decrease in the general mortality? Is it due to the increasing virulence of the disease, or because we do not treat it as successfully as our fathers?

Unfortunately the discovery of the pneumococcus, while it gave us a new theory for the disease, did not improve in the least our ability to cope with, or cure it. Since that discovery it has become pretty generally accepted that, "Pneumonia is a self-limited disease, and runs its course uninfluenced in any way by medicine. It can neither be aborted nor cut short by any means at our command. We have no specific treatment for pneumonia."

I will not consume your time by going into the etiology and pathology of this disease, for you are all familiar with both as described by Osler, Anders, Tyson and others.

PERSONAL EXPERIENCES.

You will pardon me for being personal. When I returned from college the first question my old preceptor asked was, "Would you blister in pneumonia?"

"Of course not, it is a self-limited disease, and is not affected by drugs. I would apply ice bags and give dovers powders to make them rest easy, sustain and nourish patient till the crisis came."

"Yes, and you will make many a one 'enter on eternal rest' by that treatment. You can't practice that way in my family. To be successful in treating pneumonia you must give calomel and blister."

I watched this doctor of the old school, a veritable Dr. McClure, practice for years, and his success in treating pneumonia was phenomenal. He did not lose over four per cent.

A few years ago my daughter had pneumonia and believing the blister barbarous I used other treatment for four days. When the disease began to encroach upon the upper lobe I decided to apply the blister. At that time her temperature was 104°, she was very restless, nervous and suffering intensely. The blister was applied in the afternoon, that night she slept well and next morning her temperature was 98°.

Last year I had pneumonia, and I shall never forget the relief experienced when vessication began. With this personal experience, I will continue to use calomel and blister in treating pneumonia when indicated, until a pneumonia antitoxine is discovered equal in efficacy to diphtheria antitoxine. I am not prepared to assert that the blister aborts pneumonia, but it certainly relieves the pain as nothing else does, and in my experience there has been a singular coincidence between vessication and crisis.

TREATMENT.

In order to ascertain how pneumonia is generally treated in the country, where the mortality is not so great (as admitted by the text-books), I addressed the following questions to fifty members of this association, who live outside of the larger cities. Thirty-eight replied but eleven gave no records, only a few remarks on their treatment. The following is a summary of these replies:

1. How many cases of pneumonia have you treated during the past winter? 265.

2. How many terminated by crisis? 170.

3. How many deaths? 15 or 5.7 per cent.

4. How many treated since 1900? 351. (Many kept no records.)

5. How many deaths? 21 or 5.9 per cent.

6. Do you blister? Yes 21. Rarely 4. No 2.

7. Do you use cold packs? No 24. Rarely 2. Sometimes 1.

8. Antipyretics? Yes 6. No 8. Sometimes 13.

9. Calomel? Yes 27.

10. Digitalis? Yes 17. No 3. Sometimes 8.

11. Strychnine? Yes 27. No 1.

11. Whisky or brandy? Yes 22. No 3. Sometimes 3.

13. Diet? Light but nourishing, milk, broth, soups, milk-punches, eggs, etc.

14. Any suggestions from your experience will be appreciated. These were numerous. Plenty of pure air and water, but the room should be kept about 65°. Many who used the blister preferred turpentine, mustard, or antiphlogistine on young children.

Some gave full outline of treatment throughout the disease, giving calomel, applying the fly-blister, veratrum viride, expectorants, bromides, quinine, etc., though most agreed that it was far better to treat the patient than the disease. The answers to questions 4 and 5 are very incomplete, as so many had kept no records, and could not remember cases so far back. It will be noted, however, that for those who did send in reports that appeared accurate, the mortality was practically the same as that of last winter, only 0.2 per cent. difference.

The therapeutic agents most relied on are calomel, strychnine, alcohol, blister, dovers powders, digitalis, veratrum viride, carbonate ammonium, quinine, creosote, salicylate sodium, turpentine, mustard, antiphlogistine, etc.

One doctor of over thirty years' practice and large experience had very pronounced views on the new methods of treatment. He wrote: "The man who applies ice or cold water to reduce temperature in pneumonia should not only be prosecuted for malpractice, but hung by the neck until he was dead, dead! dead!!"

The six who rarely or never blistered, two of whom sometimes used cold applications, treated 71 cases and lost 6 or 8.4 per cent. The twenty-one who blistered treated 194 cases and lost 9, or 4.6 per cent. Thus it will be seen that in the country, where we get all the fresh air needed, the mortality of those who do not blister is nearly twice as great as those who do. Another point of interest was that those who used antipyretics lost a larger per cent. than those who did not, while those who used whiskey or brandy were more successful than those who did not.

Although the blister is used so successfully by such a large proportion of country physicians, it is not mentioned or recommended by any of the recent popular text-books on practice except by Tyson, and he does it with an interrogation. "Should we blister in pneumonia? A blister in pneumonia sometimes does much good. It is specially useful in delayed resolution, late in the disease, when crisis has been imperfect and convalescence does not set in. It may even take the place of local or general blood-letting. When a blister is applied let it be a good one. A large blister is no more painful than a small one, and neither is as painful as is generally supposed."

The writer agrees with Dr. Herrick in "Reference Handbook of Medical Science," and with several papers read before this Association in 1900 and 1896. The blister should be applied at any stage, the earlier the better. The relief of pain, dysponea, cough and fever follows immediately the action of fly-blister. If the blister can be applied in twenty-four hours after the rigor, there will often be no consolidation, the disease will be arrested in the first stage, and convalescence will begin.

### HYPODERMOCLYSIS.

The hypodermic injection of from 500 to 1000 c. c. of hot (110°F.) normal saline solution is said to be a very valuable therapeutic agent. The indications for its use are shallow, irregular respiration, cyanosis, weak thready pulse or coma. It is best given in the flanks and should be repeated every hour or two if necessary. The rectal injection of salt solution is sometimes very beneficial.

Oxygen is an agent I have never had occasion to use, though many who have think their patients have been benefited by proper inhalation of this life-giving element. It is expensive and difficult to obtain outside of the large cities, so that a country doctor does not often find it in his therapeutic armamentarium.

### REST.

The importance of rest in treating pneumonia can not be overestimated. Hence the frequent administration of expectorants, nourishments, hypodermic injections, changing poultices, and a thousand and one things that suggest themselves to the anxious relatives are to be prohibited. In no disease is the assistance of a well-trained, reliable nurse more necessary, so that the patient can have rest as well as medicine and attention.

### CONCLUSIONS.

1st. The mortality from pneumonia is increasing much more rapidly in the cities than in the country.

2d. That eighty per cent. of the country physicians still use the blister, and "treat" their pneumonia cases.

3d. The therapeutic agents that give the best results are calomel, fly-blister, whiskey, dovers powders, and strychnine.

4th. That the mortality in pneumonia treated with the cold application is nearly double that treated with the blister.

LITERATURE.

American Year Book Medicine and Surgery, 1904.

Journal American Medical Association, February 28, 1903, and March 19, 1904.

Atlanta Journal-Record Medicine, April, 1904.

Anders' Practice Medicine.

Osler's Practice Medicine.

Tyson's Practice Medicine.

Reference Handbook Medical Science. Vol. IV.

